# *Cell Stress* - a new journal for cellular pathophysiology

**DOI:** 10.15698/cst2017.10.101

**Published:** 2017-10-01

**Authors:** Andreas Zimmermann, Christoph Ruckenstuhl, Katharina Kainz, Sebastian Hofer, Frank Madeo, Didac Carmona-Gutierrez, Guido Kroemer

**Affiliations:** 1 Institute of Molecular Biosciences, NAWI Graz, University of Graz, Graz, Austria.; 2 BioTechMed Graz, Graz, Austria.; 3 Equipe 11 labellisée Ligue contre le Cancer, Centre de Recherche des Cordeliers, INSERM U 1138, Paris, France.; 4 Metabolomics and Cell Biology Platforms, Gustave Roussy Comprehensive Cancer Center, Villejuif, France.; 5 Université Paris Descartes, Sorbonne Paris Cité, Paris, France.; 6 Université Pierre et Marie Curie, Paris, France.; 7 Pôle de Biologie, Hôpital Européen Georges Pompidou, Paris, France.; 8 Karolinska Institute, Department of Women's and Children's Health, Karolinska University Hospital, Stockholm, Sweden.

**Keywords:** apoptosis, autophagy, cellular stress, open access, systems biology, cancer, neurodegeneration, genome instability, DNA damage, protein misfolding, heat shock response, unfolded protein response, autophagy, lipotoxicity, mitochondria, necrosis, oxidative stress, aging, cell death, immune response, inflammation, tumor microenvironment, bacterial and viral infections, radiation

Any disease that humanity encounters is preceded by a homeostatic disruption as a prelude to a consequent pathological state. Medical researchers and practitioners try to counteract this by designing and optimizing appropriate diagnostic tools and therapeutic strategies. The European Research Institute for Integrated Cellular Pathology (ERI-CP) is one among many organizations that contribute to these efforts. ERI-CP is a virtual institute that unites highly renowned scientists from all areas of disease-oriented fundamental and clinical research. ERI-CP promotes cell biology research applied to major human diseases, including (but not limited to) neurodegenerative diseases, myopathies, mitochondriopathies, infectious diseases, cancer, and pathological aging. ERI-CP aims at establishing a continuum between fundamental, translational and clinical research. One of the organization’s major goals is to provide independent, authoritative and evidence-based advice to underpin policy for stimulating the implementation of systems biology in the investigation, classification, diagnosis and therapeutic management of major human diseases. ERI-CP aims at disseminating "official" (but ameliorable) methods and protocols applicable to the exploration of cellular pathologies. ERI-ICP also emerges as an organizing platform for international conferences and participates in efforts of data integration and distribution.

As a consequence, to provide a platform for exchanging data and ideas in the cellular pathology field, ERI-CP now launches a new journal, *Cell Stress*. This open-access, online-only journal covers all areas from basic research to therapy development and evaluation. *Cell Stress* intends to provide a platform for unraveling the link between failure to maintain homeostasis, cellular aberration and organismal pathologies. In addition, *Cell Stress* will promote the quest for diagnostic markers, as well as therapeutic interventions against a manifold of pathologies, tying basic to translational research and clinical application.

In all pathologies cellular stress is present as either a cause or a consequence. Therefore, *Cell Stress* fills a very specific niche by covering this pathology-imminent feature in all its pervasiveness and heterogeneity. This includes not only the various sources of stress, but also the corresponding cellular responses. Stress signaling cascades are omnipresent, evolutionary conserved cellular strategies to recover homeostasis and contain the onset of pathologies. Prolonged or exaggerated stress as well as the failure to mount an antagonizing response will eventually lead to the ignition of lethal signals, as often observed in neurodegenerative disorders upon proteotoxicity. Conversely, when cells evade cell death execution, cellular stress will abet the emergence of malignancies like cancer. Thus, any insight into the source, transmission, molecular targets or intrinsic and extrinsic prevention of cellular stress is of direct use for understanding and mending disease states. For this purpose, *Cell Stress* covers multifaceted topics including (but not limited to): intracellular perturbations like genome instability, DNA damage and repair, transcriptional fidelity and functional RNAs, protein (mis-)folding and quality control like the heat shock response, the unfolded protein response and autophagy, lipotoxicity, metabolic abnormalities, mitochondrial damage and oxidative stress response, aging and cell death. *Cell Stress* will also deal with multicellular stress response, such as immune responses, inflammation and the tumor microenvironment. Beyond responses to endogenous stressors, *Cell Stress* will publish papers on exogenous noxa such as bacterial/viral infections, radiation or toxic substances. Articles may also feature the use of integrative systems biology, which implements the application of "holistic" data (e.g. metabolomics, genomics, transcriptomics) as well as trans-species approaches to link ephemeral sources of stress to disease etiology and progression.

*Cell Stress* meets the challenge of handling this broad thematic diversity by a publication strategy built on several pillars. The journal uses the experience and reputation of its sister journal, *Microbial Cell*, and the dynamic technological platform of Shared Science Publishers, a young publishing house devoted to the dissemination of scientific data and knowledge as a universal public good and not a restricted pay-per-view source. Importantly, *Cell Stress* is a journal with its policies designed and its content curated by scientists, who have a broad experience in the publishing field, but who are primarily active researchers rather than specialists only committed to the publishing process itself. This is clearly mirrored at several levels, from the uncomplicated submission system to the attempt to untighten formal rigidness. In fact, *Cell Stress* implements a high degree of formal flexibility to facilitate the inclusion of all kinds of key observations in an accurate manner. Therefore, the article types offered by the journal are diverse and range from primary research articles and reports to different types of review and commentary articles, most of which are not subjected to limits in terms of word count, number of figures or references. This policy relieves the authors from the formal pressure that otherwise might force scientists to omit crucial explanations, data and references. While this policy aims at breaking restrictions, the *Cell Stress* peer review and editorial handling guidelines do also demand scientific precision in the manuscripts. Thus, referees and editors will ensure that the language of the article is clear and concise, that the figures reflect only results essential for the conclusions of the article, and that the references reflect a correct and balanced picture of the literature.

Of note, *Cell Stress* is committed to the principles of open-access publishing, making all articles freely available on the World Wide Web to be read, downloaded, stored, printed, copied, and distributed by any scientist or interested individual around the globe. To finance this policy, *Cell Stress* charges authors or research sponsors for each article published with article processing charges (APCs). This follows the concept that publishing a research work and thus relevant data and/or stimulating thoughts is an elemental part of doing research. Consequently, the costs generated from publication should be considered as one of the basic expenses to be covered by research grants or by the authors’ institutions. The last decade has witnessed this concept being embraced and translated into institutional policies as reflected in the so far almost 600 signatories of the Berlin Declaration on Open Access to Knowledge in the Sciences and Humanities. This policy echoes our conviction that the free exchange of ideas is one of the most valuable cornerstone and accelerator of scientific advancement, on the one hand, and public knowledge, on the other hand. For scientists publishing with *Cell Stress*, this open access policy maximizes the visibility of their works, promoting the quick and unrestricted use of their published data by other researchers. Therefore, *Cell Stress* authors - who retain full copyright of their work - must agree to make their articles legally available for reuse with no permissions required or fees raised, as long as they and the journal are appropriately cited as the original source. Thus, we meet our responsibility as a journal to conserve and distribute the public good that is scientific knowledge by enabling its universal online accessibility. We believe that this is especially important for works with direct pathophysiological and therapeutic worth like those published in *Cell Stress*: their technical and medical interest should be beneficial to both the scholarly and general readerships.

Finally, we find it crucial to ensure that articles published in *Cell Stress* meet the highest scientific standards. Therefore, *Cell Stress* has put together an outstanding Editorial Board comprising the most competent scientists within their area of specialty, including five Nobel Prize laureates and over 30 members of different National Academies of Science around the world. Their advice and contribution in assessing submitted papers as well as their selection of and interaction with referees will contribute considerably to drafting the best manuscripts and improving them during the revision process.

In sum, *Cell Stress* offers readers and experts all over the world a common, peer-reviewed stage to develop and transfer technical and experimental knowledge, to understand cellular and organismal stress and, ultimately, to combat disease.

